# Increasing incidence of IV‐drug use associated endocarditis in southern West Virginia and potential economic impact

**DOI:** 10.1002/clc.23162

**Published:** 2019-03-14

**Authors:** Mark C. Bates, Frank Annie, Ayan Jha, Fred Kerns

**Affiliations:** ^1^ CAMC Vascular Center of Excellence Charleston Area Medical Center Charleston West Virginia; ^2^ Center for Health Services and Outcomes Research Charleston Area Medical Center Education and Research Institute Charleston West Virginia; ^3^ Division of Policy Translation and Leadership Development Harvard T.H. Chan School of Public Health Boston Massachusetts; ^4^ Charleston Area Medical Center 3200 MacCorkle Avenue SE Charleston West Virginia

**Keywords:** drug abuse, endocarditis, heath care costs, illicit drug use, IV drug use

## Abstract

**Background:**

The opioid crisis has disproportionally affected Appalachia. One of the potentially lethal and costly complications associated with IV drug use is infective endocarditis (IE). The goal of this study was to assess the trend and costs of substance abuse associated IE admissions in Southern West Virginia.

**Methods:**

This is a retrospective analysis of cost, incidence, and geographic patterns of all patients admitted over the last decade with concomitant drug abuse (cocaine, amphetamine, sedative, and other/mixed drug abuse) and IE in the largest tertiary care center for Southern West Virginia. A time series model was used to investigate the effect of drug use on the incidence of IE.

**Results:**

A total of 462 patients were hospitalized with IE and concomitant illicit drug use. IE cases increased from 26 admissions in 2008 to 66 in 2015. Patterns of increases in mixed drug use (DRG most often associated with IV drug use in our center) mirrored increases in IE (*P* = 0.001). From 2008 to 2015, the total hospital charges were $17 306 464 on 462 cases of illicit drug associated IE. Only a fraction of the billed fees (22%) was collected ($3 829 701).

**Conclusions:**

The number of patients hospitalized with IE has dramatically increased over the last decade in a pattern that mirrors the increase in mixed drug use. The majority of payers were from underfunded state programs or private pay and thus, only 22% of the hospital charges were paid, leaving a hospital deficit of over $13 476 763 during the study period.

## 1 INTRODUCTION

Over the last decade, there has been a shift in the geographic patterns of intravenous drug use (IVDU) in the United States from focal urban locations to rural areas.^1^, ^2^ There are various socioeconomic factors that have influenced these patterns alongside more liberal opioid prescription practices.^3^ The shifting burden of IVDU to more rural areas has created unique challenges for patient access (medical attention, addiction education, and rehabilitation), as well as an avalanche of additional costs for hospitals located within the endemic areas. Coinciding with the escalation of drug‐related socioeconomic burden, the rural hospitals, and tertiary referral centers in Appalachia are caring for an increasing density of uninsured and underinsured patients.^4^, ^5^ In addition, poor sterile technique used by IVDUs and immune compromise from dietary deficiencies in drug abusers has caused an increase of hepatitis C and other related illnesses with dramatic loss of productivity, reduced long‐term quality of life, and increased healthcare costs.^6^, ^7^ The Appalachian syndemic of the opioid crisis, worsening hepatitis C, recent human immunodeficiency virus (HIV) outbreak and economic challenges is punctuated by recent data suggesting West Virginia now leads the nation in mortality from drug overdoses (35.5 deaths per 100 000).^1^

It is well known that poor sterile technique and repeated interruption of the skin/blood barrier have been implicated in IVDU associated infective endocarditis (IE) (IVDU‐IE) that most frequently involves the tricuspid valve.^8^ In addition, the use of unsterilized needles, bacteria in the injectate (drug, diluents, and fillers), can result in the introduction of bacteria or fungi directly into the bloodstream. These patients are also prone to developing immune compromise because of associated hepatitis, poor nutrition, and poor dentition. IVDUs also have higher rates of nasal and cutaneous colonization with *Staphylococcus aureus* than patients who use illicit drugs by the oral route exclusively.^9^ *S. aureus* is the most common infectious agent in these patients and IE is on the rise within the United States.^10^ Studies have analyzed differences in the effect of drug abuse on endocarditis, such as age, outcome, healthcare cost, and the patterns of geographical abuse.^11^ It is clear that IVDUs are at an increased risk for IE, have a higher incidence of recurrent IE and higher expected mortality than those who develop IE without associated drug addiction.^12^ These patients often require high‐acuity hospital resources and prolonged intensive care.^9^ There are also long recovery times from valve surgery, if needed. In addition, IVDUs‐IE often seek delayed medical attention which poses significant challenges to emergency departments and front‐line physicians.^11^ At the same time that collateral healthcare costs from the drug epidemic have been increasing, the economic downturn, and contraction of revenue from the energy sector have negatively impacted the rural healthcare systems in Appalachia amplifying the economic impact on hospitals and healthcare providers.^13^, ^14^ The crisis created by the collision of rural economic instability and the drug epidemic in Appalachia has stimulated much discussion on needed policy reform.^13^, ^14^, ^15^, ^16^, ^17^, ^18^

Our center is the largest cardiovascular tertiary referral center in southern West Virginia and resides in the epicenter of the Appalachian opiate epidemic and energy sector decline associated economic collapse. The center has seen alarming increases in IE and we hypothesized that this was due to increases in IVDU‐IE. Since IE is often very difficult to treat and consumes significant hospital resources, we set out to quantify changes in IE hospitalization patterns and associated costs in southern West Virginia.^15^, ^16^, ^19^

## 2 METHODS

This is a retrospective analysis of patients admitted to Charleston Area Medical Center (CAMC) between 2008 and 2015 with the concomitant diagnosis of IE and illicit drug use. CAMC is a large non‐profit 900 bed hospital that services around 300 000 hospital patient visits per year. The study was supported by the CAMC Health Education and Research Institute and initiated after CAMC Institutional Review Board approval. The study harvested data from the CAMC digital database utilizing ICD codes that allowed collection of data on patients that carried the diagnosis of IE and drug use during the same hospitalization. Drug use was further divided based on type of drug implicated (opioid, cocaine, amphetamine, sedative, and mixed drug abuse). Mixed drug use is the most common DRG used in our center for patients with IVDU. Specifically, the hospital data was pulled in raw form from the total number of patients billed under the DRGs of infections and drug‐related hospital admissions. Data were then transformed to patient cases in which a drug case and infection case were both charged at the same visit. These cases were then filtered by codes of endocarditis and confirmed infections and the final number was filtered to equal (N = 462). Total drug admissions were compared to identified cases of endocarditis.

The data variables obtained from the CAMC database included: Number of cases of patients hospitalized with IVDU and IE and patient variables including time of hospitalization, Zip codes, type of drug use (opiates, cocaine, amphetamines, sedatives, and mixed drug use), gender, age, race, and insurance status. Primary payers were identified by federal, state, and private payers and the study further subdivided all primary payers. We obtained detailed analysis of hospital charges to better understand the current annual economic burden for our center and federal/state funding agencies.

## 3 STATISTICAL ANALYSIS

We created a time series model in STATA 11.4 to investigate the effect of drug use on charged cases of endocarditis. A time series model was created for hospital visits involving opiate, cocaine, amphetamine, sedative, and mixed drug abuse. A multiple variable time series explored the relationship between the one data set and assessed the potential relationship between certain drug types including opiates, cocaine, amphetamine, sedative, and mixed drug abuse with the incidence of endocarditis. To create differing maps and understanding the effect of cases on regions of the state of West Virginia, Quantum GIS was used to examine these regions and to create a hot spot analysis. A hot spot analysis was constructed to understand potential areas of concern that may benefit from further investigation.

The analysis equation was filtered to include endocarditis cases that had a drug‐related charge on the same account. The equation also contains controls for the median household income and long‐term temporal trends per study year based on West Virginia Median Household Income Data (Federal Reserve Bank of St. Louis) (2008 to 2015).^20^

**Equation #1**

*Endocarditis (patients with drug‐related case)_t_ = B_1_ (exposure) _t−1_ + B_2_ (median household income) _t−2_ + B_3_ (year) _t−3_ + e (0, σ^2^)*

The exposure variables contain opiate, cocaine, amphetamine, sedative, and mixed drug abuse. The subscript “*t*” refers to the year sequence starting 2008 to 2015 for *t* − 1. Next, the time series model assumptions were verified with standard diagnostic tests which included (normal distribution, autocorrelation and partial autocorrelation, temporal autocorrelation, and Akaike's and Bayesian information criterion). A histogram was created to confirm that residuals were normally distributed of the five exposure variables. Opiates were normally distributed as well as cocaine and amphetamines. The sedative variable had a left‐skewed distribution as well as the mixed drug use exposure variable. To normalize the skew, a Shapiro‐Wilk test was used to transform the variables into a normal distribution. Autocorrelation and partial autocorrelation function did verify that five models and exposures did not contain significant temporal autocorrelation. The final test was an Akaike's and Bayesian information criterion to ensure the best fitting exposure metric and corresponding time series model. Only patients with a drug‐related case were used within this analysis this attempts to screen out potential non‐cases.

## 4 RESULTS

**Figure 1:**
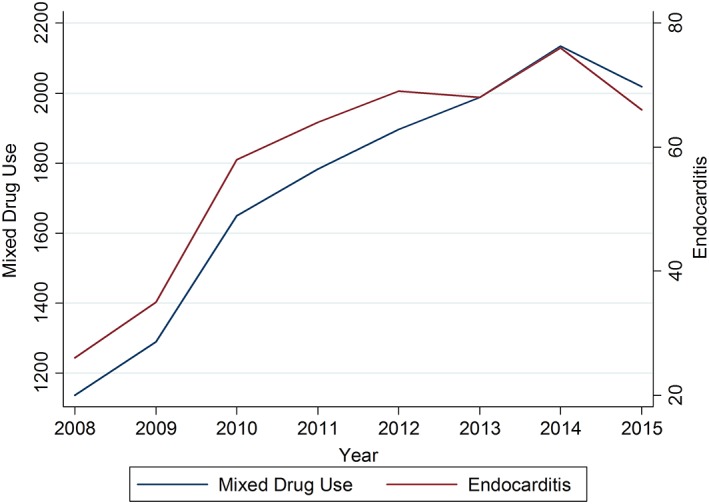
Time series analysis of mixed drug use total admissions compared to endocarditis total admissions cases over the study period 2008 to 2015

The demographics of the study population are shown in Table 1. Through the years there was a significant increase in admissions with any type of drug use as well as associated endocarditis as illustrated in Table 2. Increases in mixed drug use strongly correlated with increased cases of IE during the study period (*P* = 0.001). This suggests that for every 100 cases of mixed drug use there is a 0.06% increase in relative risk of endocarditis. This strong relationship can further be investigated with a time series line graph to illustrate this visual relationship over the same time period Figure 1. As shown within the time series line plot graph, endocarditis drug patients with mixed drug use have an almost mirrored relationship and most of the drug use related endocarditis nearing the end of the study was in the mixed drug use category.

**Table 1:** Demographic data

N = total cases	462
Sex	
Male	225
Female	237
Race/ethnicity	
White (non‐Hispanic)	429
Black (non‐Hispanic)	29
Other/unknown	4
Age (years)	
16‐29	102
30‐39	159
40‐49	94
50‐59	74
60‐69	26
70‐85	7

**Table 2:** Results of times series analysis of (exposure variables) use on total admissions of (endocarditis)

Variable	Coefficient	SE	T	*P > t*	95% confidence interval
Model 1					
Mixed drug use	0.06475	0.0061	10.56	0.001	0.0477‐0.0817
Median income	0.0061	0.0037	1.64	0.176	−0.0042‐0.0165
Year	−0.0225	0.0093	−2.42	0.073	−0.0484‐0.0033
Model 2					
Opiates	−0.0002	0.0001	−1.18	0.304	−0.0007‐0.0003
Median income	−0.0001	0.0001	−0.99	0.377	−0.0001‐0.0001
Year	0.0897	0.0247	3.63	0.022	0.0210‐0.1583
Model 3					
Cocaine	−0.0018	0.0025	−0.73	0.508	−0.0091‐0.0053
Median income	−0.0001	0.0001	−1.09	0.336	−0.0003‐0.0001
Year	0.0728	0.0179	4.06	0.015	0.0230‐0.1226
Model 4					
Amphetamine	−0.0013	0.0011	−1.09	0.335	−0.0046‐0.0020
Median income	−0.0001	0.0001	−1.19	0.300	−0.0002‐0.0001
Year	0.0834	0.0214	3.88	0.018	0.0237‐0.1431
Model 5					
Sedatives	0.0013	0.0013	1.03	0.362	−0.0023‐0.0051
Median income	−0.0007	0.0000	−0.96	0.393	−0.0002‐0.0001
Year	0.0804	0.0202	3.98	0.016	0.0242‐0.1365

The overall state health metric variable of household income was not significantly associated with the increase in endocarditis (*P* = 0.176). The payer mix for the study cohort is detailed in Table 3 alongside all the hospital charges vs the actual payment received. Private pay and the WV Heath and Human resources Medical Card made up a significant component of cases. The financial impact on Federal and State systems was dwarfed by the economic burden on the hospital as reflected by the low proportion of payments (22% of overall charges) received. This translates to over $13 million in losses over the entire study period. More importantly, over $2.3 million of losses were seen in the last year of the study alone.

**Table 3:** Primary payers

Insurance status/charged payers	N	Total collected	Total charged
West Virginia Health Human Services Medical Card	237	$1 570 617	$9 375 671
Self‐pay	40	$0	$971 751
Blue Cross Blue Shield	27	$887 117	$1 141 537
Medicare	27	$477 262	$1 748 267
West Virginia Medicaid	26	$120 779	$820 890
Medicare Part B	22	$58 056.89	$293 554.61
Unicare	17	$170 025	$879 029
Medicaid	23	$120 793	$862 009
Charity	8	$0	$111 945
Health smart/public employee insurance agency	5	$13 194	$108 265
Other insurances	30	$466 675	$ 1 268 052
Total	462	$3 829 701	$17 306 464

**Figure 2:**
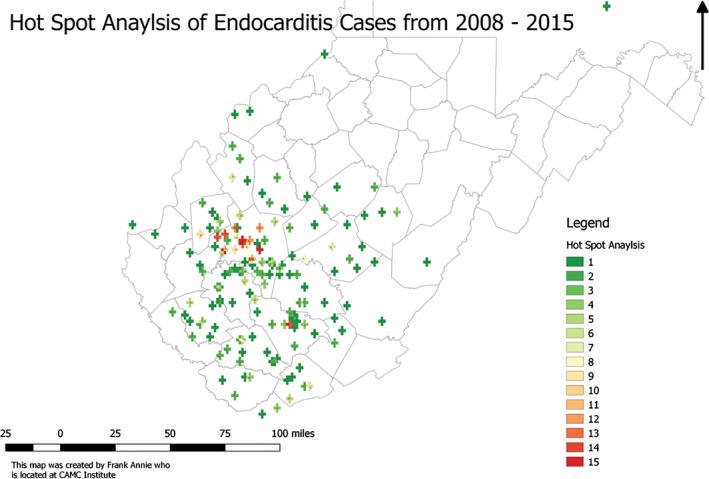
Hot spot analysis of endocarditis cases in Southern West Virginia 2008 to 2015

The geographic analysis of the patient origin, based on admission home address zip code, showed a pattern of clusters within the major population centers surrounding coalfields as highlighted in Figure 2. These areas in southern West Virginia have been most impacted by the collapse of the energy sector and associated persistent economic contraction.

## 5 DISCUSSION

IE admissions to the largest southern West Virginia tertiary care hospital have more than doubled over the last decade and this disturbing trend mirrors the pattern of increased mixed drug use seen in this patient population. This study provides insight into the IVDU‐IE component of the Appalachian opioid syndemic. The paper complements the recently published American Enterprise Institute (AEI) analysis of geographic variation in the costs of the opioid crisis that suggested West Virginia loses $8.8 billion annually from the opiate epidemic.^19^ The AEI report estimates the overall cost of this syndemic is $4793 per person living in West Virginia . While IVDU‐IE is a small component of the Appalachian opioid crisis syndemic cost, the annual hospital losses exceeded $480 000 in 2008 and accelerated to $2.3 million in 2015 suggesting this is a real crisis for the hospital. Our medical center has also experienced contracting reimbursement and unprecedented increases in charity care over the last decade for patients beyond those analyzed in this study. For example, only 17.4% of CAMC reimbursement comes from private insurers compared to the 34% national average reported in 2016.^21^

Our findings associating the opioid epidemic with increased IE mirrored the findings reported by our colleagues at the University of Kentucky (UK). UK is the largest tertiary care center supporting eastern Kentucky and that region happens to also reside on the opioid crisis syndemic fault‐line. They found that the number of IE cases doubled from 1999 to 2015.^12^ The findings reported herein also complement observations from a retrospective 6‐year analysis of the North Carolina Hospital Discharge Database. The North Carolina study design was different, making outcomes difficult to compare, but over the 6‐year study period they noted a 12‐fold increase in hospitalizations with “drug addiction and IE” with an annual cost increase from $1 million per year to $22.2 million per year.^12^, ^22^ Hartman et al reported a similar series to ours based on a retrospective analysis of 127 patients in a single North Carolina tertiary care hospital. They found incident admissions for IE and associated IVDU increased from 14% of all IE patients to 56% from 2009 to 2014.^16^, ^22^ In addition, other studies have shown that serious infections associated with IVDU have also increased significantly with associated costs in 2012 of $700 million in the United States.^23^, ^24^ It should be noted that the increase in IVDU‐IE is not isolated to Appalachia with less dramatic trends also reported in differing regions of the country.^22^, ^23^, ^24^, ^25^, ^26^, ^27^, ^28^

The average charges for our IVDU‐IE patients in the last year of the study (approximately $70 000/patient) are well below the national average of $120 000 and may reflect our team's attempts to avoid open heart surgery since IVDU‐IE patients are 10 times more likely to die or need a second surgery.^16^, ^28^

This study has many limitations. First, in our center, the diagnosis of “mixed drug use” strongly correlates with IV drug use but it is not definitive. We elected to use this DRG since often these patients deny IVDU and clear objective clinical signs are often lacking. Also, the use of hospital ICD9/10 codes to establish a diagnosis is associated with several known limitations. Thus, the findings must be considered in that context. As a countermeasure for these limitations, patient data and drug use data were combined to filter out other cases of endocarditis that did not appear to have a drug‐related cause. This approach allows for multiple filters to view the relationship that is occurring in the region; unfortunately, this method may under report certain cases. We acknowledge these limitations and accordingly describe the findings as associations without concluding causation. Also, the review of echocardiograms and definition of organism types would have further strengthened the conclusions by providing another layer filter.

## 6 CONCLUSION

This study highlights how a previously rare life‐threatening infection, IE, has dramatically increased in the wake of the Appalachian opioid epidemic. While the number of patients with IVDU‐IE are eclipsed by the growing list of other opioid syndemic diseases (hepatitis C, HIV, and overdose), the costs per patient and overall healthcare system impact cannot be underestimated. The increase in IVDU‐IE in southern West Virginia comes at a time when the region faces an unrelenting recession and after hospital resources have been extinguished by contracting reimbursements and regional economic challenges.^17^ The Southern West Virginia energy sector contraction and associated unemployment persists, in part, because of concerns from new industries regarding relocation to a region with such a high incidence of opiate dependence in a potential workforce. This economic downturn cycle along‐side increases in prescription opioid medications and influx of inexpensive heroin, have all likely participated in the regional opioid crisis.^19^, ^29^

Further research is needed to understand the overall impact of illicit drug use in the region and the multiple socioeconomic issues or other causation variables that may be participating in the epidemic. However, based on the findings reported herein, investment in resources for high‐risk areas, defined by geo‐map driven hot spot analysis, for needle exchanges and rehabilitation centers may be warranted. Along those lines, we are now seeking to define clusters or regions with increased serious infection risk so we can study the impact of local interventions and hopefully translate these findings into preventive solutions.

## CONFLICT OF INTEREST

The authors declare no potential conflict of interests.

## References

[1] Rudd RA , Aleshire N , Zibbell JE , et al. Increases in drug and opioid overdose deaths—United States, 2000‐2014. MMWR Morb Mortal Wkly Rep. 2016;64:1323‐1327. 10.15585/mmwr.mm6450a3 26720857

[2] Ratcliffe M , Burd C , Holder K , Fields A . Defining rural at the US Census Bureau. Washington, DC: *American Community Survey And Geography Brief*; 2016:1‐9.

[3] Unick G , Rosenblum D , Mars S , Ciccarone D . The relationship between US heroin market dynamics and heroin‐related overdose, 1992‐2008. Addiction. 2014;109:1889‐1898. 24938727 10.1111/add.12664 PMC5725335

[4] Cooper HL , Brady JE , Ciccarone D , Tempalski B , Gostnell K , Friedman S . Nationwide increase in the number of hospitalizations for illicit injection drug use‐related infective endocarditis. Clin Infect Dis. 2007;45:1200‐1203. 17918083 10.1086/522176 PMC2567828

[5] Rossen LM , Khan D , Warner M . Hot spots in mortality from drug poisoning in the United States, 2007‐2009. Health Place. 2014;26:14‐20. 24333939 10.1016/j.healthplace.2013.11.005 PMC4659494

[6] Gordon SC , Elloway RS , Long JC , Dmuchowski CF . The pathology of hepatitis C as a function of mode of transmission: blood transfusion vs. intravenous drug use. Hepatology. 1993;18:1338‐1343. 8244258

[7] Barocas JA , Brennan MB , Hull SJ , Stokes S , Fangman JJ , Westergaard RP . Barriers and facilitators of hepatitis C screening among people who inject drugs: a multi‐city, mixed‐methods study. Harm Reduct J. 2014;11(1):1. 24422784 10.1186/1477-7517-11-1 PMC3896714

[8] Hussain ST , Witten J , Shrestha NK , Blackstone EH , Pettersson GB . Tricuspid valve endocarditis. Ann Cardiothorac Surg. 2017;6:255‐261. 28706868 10.21037/acs.2017.03.09 PMC5494428

[9] Olubamwo O , Onyeka IN , Aregbesola A , et al. Association between route of illicit drug administration and hospitalizations for infective endocarditis. SAGE Open Med. 2017;5:205031211774098. 10.1177/2050312117740987 PMC5734445 29276587

[10] Chatterjee SS , Otto M . Improved understanding of factors driving methicillin‐resistant Staphylococcus aureus epidemic waves. Clin Epidemiol. 2013;5:205‐217. 23861600 10.2147/CLEP.S37071 PMC3707418

[11] Wurcel AG , Anderson JE , Chui KKH , et al. Increasing infectious endocarditis admissions among young people who inject drugs. Open Forum Infect Dis. 2016;3. 10.1093/ofid/ofw157 PMC5084714 27800528

[12] Seratnahaei A , Leung SW , Charnigo RJ , Cummings MS , Sorrell VL , Smith MD . The Changing ‘Face’ of Endocarditis in Kentucky: an increase in tricuspid cases. Am J Med. 2014;127:786‐781. 10.1016/j.amjmed.2014.04.009 PMC4384329 24769025

[13] House of Representatives . 115th Cong 1 : The Opioid Epidemic in Appalachia: Addressing Hurdles to Economic Development in the Region: Hearings before the Subcommittee on Economic Development of Public Building, and Emergency Management. 2017.

[14] Slipczuk L , Codolosa JN , Davila CD , et al. Infective endocarditis epidemiology over five decades: a systematic review. PLoS One. 2013;8:e82665. 24349331 10.1371/journal.pone.0082665 PMC3857279

[15] Tookes H , Diaz C , Li H , Khalid R , Doblecki‐Lewis S . A cost analysis of hospitalizations for infections related to injection drug use at a county safety‐net hospital in Miami, Florida. PloS One. 2015;10:0129360. 10.1371/journal.pone.0129360 PMC4468183 26075888

[16] Fleischauer AT , Ruhl L , Rhea S , Barnes E . Hospitalizations for Endocarditis and Associated Health Care Costs Among Persons with Diagnosed Drug Dependence—North Carolina, 2010‐2015. MMWR Morb Mortal Wkly Rep. 2017;66:569‐573. 28594786 10.15585/mmwr.mm6622a1 PMC5720243

[17] Bloch H , Rafiq S , Salim R . Economic growth with coal, oil and renewable energy consumption in China: Prospects for fuel substitution. Economic Modelling. 2015;44:104‐115.

[18] Richardson LJ , Cleetus R , Clemmer S , Deyette J . Economic impacts on West Virginia from projected future coal production and implications for policymakers. Environ Res Lett. 2014;9:024006.

[19] Brill A , Ganz S . The Geographic Variation in the Cost of the Opioid Crisis. Washington, DC: American Enterprise Institute; 2018.

[20] U.S. Bureau of the Census , Estimate of Median Household Income for West Virginia. (2018). https://fred.stlouisfed.org/series/MHIWV54000A052NCEN, [MHIWV54000A052NCEN], retrieved from FRED. Accessed: March 27, 2018.

[21] NHE‐Fact‐Sheet. (2018). https://www.cms.gov/Research‐Statistics‐Data‐and‐Systems/Statistics‐Trends‐and‐Reports/NationalHealthExpendData/NHE‐Fact‐Sheet.html. Accessed: April 17, 2018.

[22] Hartman L , Barnes E , Bachmann L , Schafer K , Lovato J , Files DC . Opiate injection‐associated infective endocarditis in the southeastern United States. Am J Med Sci. 2016;352:603‐608. 27916215 10.1016/j.amjms.2016.08.010 PMC5830130

[23] Ronan MV , Herzig SJ . Hospitalizations Related To Opioid Abuse/Dependence And Associated Serious Infections Increased Sharply, 2002‐12. Health Aff Millwood. 2016;35:832‐837. 27140989 10.1377/hlthaff.2015.1424 PMC5240777

[24] Tempalski B , Pouget ER , Cleland CM , et al. Trends in the population prevalence of people who inject drugs in US metropolitan areas 1992‐2007. PloS One. 2013;8:e64789. 23755143 10.1371/journal.pone.0064789 PMC3673953

[25] Deo SV , Raza S , Kalra A , et al. Admissions for infective endocarditis in intravenous drug users. J Am Coll Cardiol. 2018;71:1596‐1597. 29622169 10.1016/j.jacc.2018.02.011

[26] Kim JB , Ejiofor JI , Yammine M , et al. Surgical outcomes of infective endocarditis among intravenous drug users. J Thorac Cardiovasc Surg. 2016;152:832‐841. 27068439 10.1016/j.jtcvs.2016.02.072

[27] Wilson LE , Thomas DL , Astemborski J , Freedman TL , Vlahov D . Prospective study of infective endocarditis among injection drug users. J Infect Dis. 2002;185:1761‐1766. 12085322 10.1086/340827

[28] Sousa C , Botelho C , Rodrigues D , Azeredo J , Oliveira R . Infective endocarditis in intravenous drug abusers: an update. Eur J Clin Microbiol Infect Dis. 2012;31:2905‐2910. 22714640 10.1007/s10096-012-1675-x

[29] Madras BK . The Surge of Opioid Use, Addiction, and Overdoses: Responsibility and Response of the US Health Care System. JAMA Psychiat. 2017;74:441‐442. 10.1001/jamapsychiatry.2017.0163 28355456

